# Depression and Social Anxiety Predict Internet Use Disorder Symptoms in Children and Adolescents at 12-Month Follow-Up: Results From a Longitudinal Study

**DOI:** 10.3389/fpsyg.2021.787162

**Published:** 2021-12-13

**Authors:** Katharina Leo, Sonja Kewitz, Lutz Wartberg, Katajun Lindenberg

**Affiliations:** ^1^Department of Psychotherapy for Children and Adolescents, Institute for Psychology, Goethe University Frankfurt, Frankfurt am Main, Germany; ^2^Department of Psychology, Faculty of Human Sciences, MSH Medical School Hamburg, Hamburg, Germany

**Keywords:** depression, social anxiety, Internet use disorders, gaming disorder, predictors, children and adolescents, longitudinal study

## Abstract

Trajectories of internalizing disorders and behavioral addictions are still largely unknown. Research shows that both disorders are highly comorbid. Previous longitudinal studies have focused on associations between internalizing disorders and behavioral addictions using screening instruments. Our aim was to develop and examine a theory-based model of trajectories, according to which internalizing disorders foster symptoms of Internet use disorders, mediated by a reward deprivation and maladaptive emotion regulation. We applied clinically relevant measures for depression and social anxiety in a prospective longitudinal study with a 12-month follow-up investigation. On the basis of an at-risk population of 476 students (mean age = 14.99 years, *SD* = 1.99), we investigated the predictive influence of clinically relevant depression and social anxiety at baseline (t1) on Internet use disorder symptoms at 12-month follow-up (t2) using multiple linear regression analyses. Our results showed that both clinically relevant depression and social anxiety significantly predicted symptom severity of Internet use disorders one year later after controlling for baseline symptoms of Internet use disorders, gender and age. These results remained robust after including both depression and social anxiety simultaneously in the model, indicating an independent influence of both predictors on Internet use disorder symptoms. The present study enhances knowledge going beyond a mere association between internalizing disorders and Internet use disorders. To our knowledge, this is the first study investigating clinically relevant depression and social anxiety to predict future Internet use disorder symptoms at 12-month follow-up. In line with our model of trajectories, a significant temporal relationship between clinically relevant internalizing disorders and Internet use disorder symptoms at 12-month follow-up was confirmed. Further studies should investigate the mediating role of reward deprivation and maladaptive emotion regulation, as postulated in our model. One implication of these findings is that clinicians should pay particular attention to the increased risk of developing behavioral addictions for adolescents with depression and social anxiety.

## Introduction

In clinical practice we notice a rising number of adolescents that show a problematic use of Internet applications. This problematic use often appears on top of other mental health issues adolescents initially seek psychological advice for.

Current research shows: There has been an increase within youth in time spent online during the past decades, which has further been accelerated by the COVID-19 pandemic [Dak-Gesundheit (Ed.), 2020; Medienpädagogischer Forschungsverbund Südwest, 2020]. An increase in time spent online increases the risk of developing behavioral addictions, such as Internet use disorders (Király et al., 2020; Rumpf et al., 2020).

Internet use disorder is not yet a formal diagnosis and consistent diagnostic criteria are still missing (Spada, 2014). However, Internet use disorder is typically characterized by symptoms of loss of control, giving priority to Internet use over other activities, and continuation despite negative consequences, which cause significant distress and impairment (e.g., Starcevic and Aboujaoude, 2017). It is further suggested as an umbrella construct for Internet-related disorders, as it comprises both the addictive use of game applications (such as video games) as well as the addictive use of non-gaming Internet applications (such as social media, online pornography, or online shopping) (Lindenberg et al., 2020). As a result of years of research on the tentative DSM-5 diagnosis of Internet gaming disorder (American Psychiatric Association, 2013), gaming disorder has been included in the new category of behavioral addictions in ICD-11 (World Health Organization, 2018). Other non-gaming activities such as social network use disorders, pornography use disorders or shopping disorders can be classified as other behavioral addictions.

Internet use disorders are already highly prevalent and prevalence estimates in representative samples of German adolescents ranged from 3.2 to 4.7% (Rumpf et al., 2014; Wartberg et al., 2015, 2017). Results from a large high-school study of *N* = 6,487 adolescents from 41 schools in Germany showed a prevalence of 6.1%, which increased from 2.8% in 11–12 year-olds to 9.1% in 18–21 year-old adolescents (Lindenberg et al., 2018). This might be of high relevance for those youth who are already struggling with psychological health. Past studies indicate a strong association between Internet use disorders and both internalizing and externalizing disorders (e.g., Carli et al., 2013; Masi et al., 2020; Paschke et al., 2020). The most frequently mentioned mental illnesses associated with Internet use disorders are ADHD, depression, and anxiety disorders with social anxiety in particular (Taranto et al., 2015; Lindenberg et al., 2017b; So et al., 2017; Franke et al., 2018; Masi et al., 2020; Wartberg and Kammerl, 2020). The associations between Internet use disorders and internalizing disorders is subject matter in the present study, as (social) anxiety as well as depression rank among the most common mental disorders in German children and adolescents (Beesdo et al., 2007; Klasen et al., 2017). For depressive symptoms, for example, a prevalence of 8.2% was reported in a representative sample of 12- to 17-year-olds (Wartberg et al., 2018a). Relations between internalizing disorders and Internet use disorders have been widely detected in cross-sectional studies (Lindenberg et al., 2017b; Masi et al., 2020). Internalizing disorders and Internet use disorders may be interlinked by common pathomechanisms of reward processing and emotion regulation (Lindenberg and Holtmann, in press; Reilly et al., 2020; Wartberg and Lindenberg, 2020). According to assumptions of clinical models of depression, a loss of positive reinforcement is assumed to play a crucial role in the development of depression. Beck’s (1986) cognitive theory of depression stresses out the role of maladaptive emotion regulation, caused by negative cognitions, in the emergence and maintenance of depressive disorders. As a dysfunctional emotional regulation strategy, social withdrawal is associated with a loss of positive reinforcement and the latter seems to represent a significant cause and maintenance factor of depression (Lewinsohn, 1974). Regarding social anxiety disorder, individuals seem to show low reward expectations about social interactions (Clark and Wells, 1995), leading to the avoidance of such situations. As a significant cause and maintenance factor (e.g., Givon-Benjio et al., 2020), avoidance behavior will eventually result in social withdrawal and leave individuals with reward deficiency of social interactions.

Emotion regulation and the role of reward processing also seems to play a significant role in the development of behavioral addiction (Lindenberg et al., 2020). As suggested subordinate category of Internet use disorders, gaming disorder seems to be strongly associated with maladaptive emotion regulation, representing a highly maintenance factor for problematic Internet use (Lindenberg and Holtmann, in press; Kökönyei et al., 2019; Lindenberg et al., 2020). It is assumed that an excessive Internet use increases individual reward sensitivity and expectation, leading to a reduction in the way a reward is being experienced in the long term (Lindenberg and Holtmann, in press; Wölfling et al., 2013; Zheng et al., 2019). As youths spent more time online and show an increase in using digital media in general, resources for reward experience are shifted from real-life to online activities. This will slowly lead to a prioritization of gaming or Internet activities over other activities (which is one core symptom of Internet use disorders). Due to mere repetition of behavior (frequent Internet use) a new habit is being built to gain short-term reward (gratification). The individual will learn to use the Internet to regulate negative emotions and compensate reward deficiency (Lindenberg et al., 2020).

First longitudinal studies have investigated the timely course between both disorder categories, however, with heterogenous findings (Ko et al., 2009; Cho et al., 2013; Strittmatter et al., 2016). The majority of the few existing longitudinal studies further operationalized internalizing disorders via subscales of screening instruments (Cho et al., 2013; Gámez-Guadix, 2014; Wartberg et al., 2021). Moreover, to date there are no theoretical models to explain the associations and trajectories of these disorders.

**FIGURE 1:**
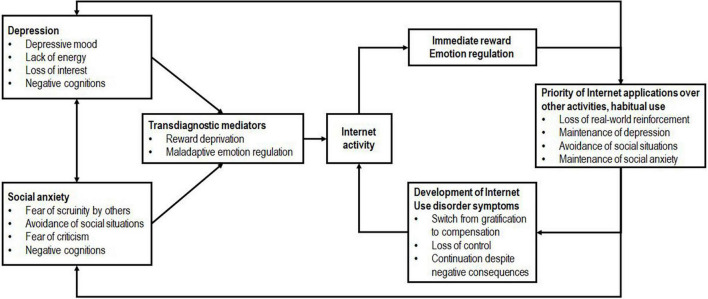
Trajectories of depression and social anxiety fostering Internet use disorder symptoms.

To fill this theoretical gap, we developed a model describing trajectories that lead from internalizing disorders to behavioral addictions. These behavioral addictions reinforce and maintain underlying depressive and social anxiety symptomatology. We assume clinically relevant depression as well as clinically relevant social anxiety to foster symptoms of behavioral addictions, Internet use disorders in particular. Despite depression and social anxiety disorder being highly comorbid, we believe that both disorders foster the development of behavioral addictions independently (see Figure 1).

Maladaptive reward processing seems to be a common factor associated with both internalizing disorders and behavioral addictions. Due to reward deficiency in real-life, and the lack of alternative, functional coping strategies we hypothesized that individuals suffering from depression or social anxiety disorder may especially experience Internet activities to be highly effective in regulating their negative emotions. The internet activity offers an immediate, consistent, and strong reward as well as a negative reinforcement by reducing emotional distress. This process is effortless, always available and keeps a safe social distance to others. Since comparable rewards in real-life (social or physical activities) are a lot harder to achieve, affected persons become increasingly less motivated to strive for such rewards. Consequently, this enhances the probability to prioritize Internet use to regain and retain an inner psychological balance. This maintains or increases both depressive symptoms (due to loss of real-world reinforcement) and social anxiety symptoms (due to avoidance of real-world social interactions). Moreover, with increased habitual use of Internet activity to regulate emotions, the initial gratification experience will shift to a compensation experience (Brand et al., 2019) and cause craving symptoms. This liking versus wanting mechanism has repeatedly been demonstrated in neurobiological studies focusing on behavioral addictions (Lindenberg and Basten, 2021). Following the cognitive behavioral etiology model of PROTECT [German acronym for “Professioneller Umgang mit technischen Medien” (Lindenberg et al., 2020)], an increased use of online applications may eventually result in a vicious circle. In this vicious circle individuals spend increasingly more time online to escape from negative consequences, leading to the development of Internet use disorder symptoms (i.e., loss of control, continuation despite negative consequences).

Current state of international literature supports these assumptions. There is evidence for disorder specific characteristics, such as difficulties in emotion regulation, to be highly associated with Internet use disorders and gaming disorder (Strittmatter et al., 2016; Kökönyei et al., 2019; Lindenberg et al., 2020; Wartberg and Lindenberg, 2020; Wartberg et al., 2021). Further findings suggest depression and social anxiety to foster the development of Internet use disorders (Ko et al., 2009; Gámez-Guadix, 2014; Vadher et al., 2019). Vice versa there are studies suggesting Internet usage to predict emotional distress (e.g., Wartberg et al., 2018b). Yet, these studies often lack clinically relevant measures and samples. Thus, the aim of the present study was to detect depression and social anxiety through clinically relevant measures in an at-risk sample of children and adolescents. We also examined to what extent each of the internalizing disorders can predict an increase in Internet use disorder symptomatology at 12-month follow-up. On the basis of our proposed model of trajectories (see Figure 1), our expectations were that (a) clinically relevant symptoms of depression predict an increase in problematic Internet use one year later in a group of children and adolescents already at risk of developing Internet use disorders, (b) within the same sample, clinically relevant symptoms of social anxiety predict an increase in problematic Internet use one year later, and (c) both disorders predict an increase in symptoms of Internet use disorders within the same sample beyond the joint share.

## Materials and Methods

### Procedure

Data collection took place within the scope of two longitudinal studies: An indicated prevention study [PROTECT study; ClinicalTrials.gov:NCT02907658 (Lindenberg et al., 2017a, submitted)] and an early intervention study [PROTECT+ study; ClinicalTrials.gov: NCT03582839 (Szász-Janocha et al., 2020a,b)]. The ethics votes for both investigations were obtained by the Research Ethics Committee of the University of Education Heidelberg (Az.: 7741.35-13), along with an approval by the Regional Council (Az.: 71c2-6499.25). Participants and parents of both surveys signed an informed written consent. PROTECT data was collected between October 2015 and September 2018 by trained psychologists in a total of 33 schools in the three federal states of Baden-Wuerttemberg, Hesse and Rhineland-Palatinate during regular school hours. PROTECT+ data was gathered during April 2016 and December 2017 by trained psychologists at the University of Education in Heidelberg and the addiction advice center of the Caritas in Mannheim. Data from both studies were merged and the results controlled for treatment condition. For the present investigation, depression and social anxiety data of the first measurements (t1) were used as predictors for Internet use disorder symptoms 12 months later (t2).

### Participants

The study population included *N* = 476 adolescents at elevated risk of developing Internet use disorders, either identified by initial screening [PROTECT study (Lindenberg et al., 2017a)] or self-selection [PROTECT+ study (Szász-Janocha et al., 2020a)]. The instrument used for screening was the Compulsive Internet Use Scale [CIUS (Meerkerk et al., 2009; Gürtler et al., 2015)], which was employed once at t1. Participants with a score higher than 20 were included. In total, the participants included in the study at hand reached elevated scores with a CIUS mean score of *M* = 27.28 (*SD* = 6.40) at baseline. The average score of Internet use disorder symptoms was *M* = 14.06 (*SD* = 7.35) as measured by our modified version of the CSAS-J [German: Computerspielabhängigkeitsskala Selbstauskunft durch Jugendliche; translation: Video Game Dependency Scale self-report by youth; (Rehbein et al., 2015)] at t1, taking both problematic gaming and Internet activities into account. Mean age of the merged sample was 14.99 years (*SD* = 1.99) and 50.0% were female at t1. Overall, 134 youths of our sample showed clinically relevant depression at baseline and 90 participants showed clinically relevant social anxiety at baseline (t1). In total at t1, 12.4% of the sample attended school at a low educational level, 21.0% at medium educational level, and 66.6% at high educational level.

### Measures

To assess clinically relevant depressive symptoms at t1, we used the German version of the Children’s Depression Inventory [DIKJ; (Stiensmeier-Pelster et al., 2014)]. The DIKJ consists of 26 items with three pre-formulated answers each (scores 0 to 2; i.e., item 1: “I rarely felt bad” for score 0, “I often felt bad” for score 1, and “I felt bad all the time” for score 2). The sum score shows the severity of depressive disorder symptoms, whereas higher values represent higher levels of depressiveness. In the present sample, internal consistency was high (Cronbach’s α = 0.84) for the DIKJ total score at baseline. According to the DIKJ manual (Stiensmeier-Pelster et al., 2014), a clinically relevant depression score applies for a *T* value of 60 or higher. We divided the sample into two groups. Participants who met clinically relevant depression at baseline were coded with “1” and all other persons with “0.”

Clinically relevant symptoms of social anxiety at t1 were determined via the German version of the Social Interaction Anxiety Scale [SIAS; (Stangier et al., 1999)]. The SIAS comprises 20 items (i.e., item 17: “I always think that when I talk I might say something embarrassing.”) on a five-point Likert scale (scores 0 = “not applicable at all” to 4 = “very strongly applicable”), to assess anxiety levels in social situations. Higher sum scores indicate higher levels of anxiety concerning social situations. The SIAS shows high discriminatory validity. Based on discrimination analysis a correct distinction of healthy people has been reached for 90% and 80% of people suffering from other anxiety disorders in the original study (Stangier et al., 1999). Thus, it represents a suitable instrument to identify participants with clinically relevant symptoms of social anxiety. In the present sample, the SIAS showed a high internal consistency with a Cronbach’s alpha of 0.89. According to Stangier et al. (1999) a SIAS cut-off score of ≥30 appears suitable to distinguish patients with social anxiety from comparative samples. Thus, we utilized this cut-off criterion to identify participants reporting a clinically relevant symptomatology of social anxiety. Again, we divided the sample into two groups. Participants who showed clinically relevant social anxiety at baseline were coded with “1” and all other persons with “0.”

The severity of Internet use disorder symptoms at baseline and 12-months follow-up was measured by the total score of the adapted Video Game Dependency Scale [Ref: German “Computerspielabhängigkeitsskala für Jugendliche”; CSAS-J (Rehbein et al., 2015)]. Items were modified to cover Internet use disorders in general, i.e., gaming disorder and Internet use disorders due to non-gaming activities (e.g., item 1: “Even when I am not gaming/online, I think about gaming/going online” to capture *preoccupation* criteria) with permission by the publisher. Previous research indicated non-gaming Internet use disorders to show similar impairments as gaming disorder (Strittmatter et al., 2015). For this reason, we considered an instrument assessing DSM-5 criteria as an appropriate measure for Internet use disorder symptoms. The CSAS comprises all 9 diagnostic criteria for Internet Gaming Disorder as defined in DSM-5. Criteria are assessed by 2 items each (18 in total). Answers are rated on a 4-point Likert scale from 0 to 3 (“strongly disagree,” “somewhat disagree,” “somewhat agree,” “strongly agree”). Scores can be interpreted dimensional or categorical. Regarding dimensional scoring, higher values display a more severe symptomatology. The CSAS-J has been validated for youths. It further shows a high face validity due to its proximity to the diagnostic criteria of Internet Gaming Disorder (Rehbein et al., 2015) and a high reliability (Cronbach’s α = 0.88) in our present sample. All measures mentioned above were carried out as self-report questionnaires.

### Statistical Analyses

The objective of our investigation, was to determine if clinically relevant symptoms of depression and social anxiety could predict Internet use disorder symptoms at a 12-month follow-up. Therefore, bivariate correlation analyses and three multiple linear regression analyses were calculated to predict Internet use disorder symptoms (CSAS-J total score at 12-months follow-up, t2) by the presence of clinically relevant depression (DIKJ) and social anxiety (SIAS) at baseline (t1). Our independent variables regarding depression and social anxiety were dichotomized with 0 = “no clinically relevant symptomatology” and 1 = “clinically relevant symptomatology.”

First, linear regression models were calculated including depression (Model 1) and social anxiety (Model 2) separately to predict Internet use disorder symptoms after controlling for age, gender and Internet use disorder baseline symptoms. Afterwards, all independent variables were simultaneously included in a multivariable regression model (Model 3). Age, gender and Internet use disorder baseline symptoms were included as control variables. All statistical evaluations were carried out with the statistical software SPSS version 27.0 (IBM, 2020, New York, NY, United States).

## Results

### Descriptive Statistics

In Table 1 bivariate correlations for all included variables were presented. Both social anxiety and depression at t1 were statistically significantly correlated with Internet use disorder symptoms at t2 (*r* = 0.26; *p* < 0.01). Furthermore, data depict a significant positive correlation for Internet Use disorder symptoms at t1 and t2 (*r* = 0.46; *p* < 0.01). Correlated data further display a negative association between gender and Internet use disorder symptoms at t1 (*r* = −0.12; *p* < 0.01), indicating a tendency in males to show more symptoms of Internet use disorder compared to females at baseline. Internet use disorder at t1 positively correlated with depression (*r* = 0.20; *p* < 0.01) and social anxiety (*r* = 0.23; *p* < 0.01), displaying a significant association of our predictors and Internet use disorder symptoms already at baseline. Depression and gender were significantly positively correlated (*r* = 0.19; *p* < 0.01), suggesting a tendency of more females inhering clinically relevant depressive symptoms in our sample. Both depression and social anxiety measures were also correlated (*r* = 0.41, *p* < 0.01).

**TABLE 1:** Pearson correlation matrix for all included variables.

	1.	2.	3.	4.	5.	6.
1. Gender (t1)	1.00					
2. Age (t1)	0.03	1.00				
3. Internet use disorder symptoms (t1)	−0.12**	–0.05	1.00			
4. Depression^1^ (t1)	0.19**	0.04	0.20**	1.00		
5. Social anxiety^1^ (t1)	0.07	0.08	0.23**	0.41**	1.00	
6. Internet Use Disorder symptoms (t2)	–0.06	–0.07	0.46**	0.26**	0.26**	1.00

**Correlation statistically significant at p < 0.01.

*^1^Clinically relevant symptoms.*

### Linear Regression Analyses

In the first regression analysis (Model 1), we found clinically relevant symptoms of depression (t1) to predict symptoms of Internet use disorder at 12-month follow-up (see Table 2) after controlling for age, gender and baseline Internet use disorder symptoms. In the second regression analysis (Model 2), clinically relevant symptoms of social anxiety (t1) predicted symptoms of Internet use disorder at t2 (see Table 2). In the third regression analysis (Model 3, see right column of Table 2), both clinically relevant symptoms of depression (t1) and social anxiety (t1) predicted symptoms of Internet use disorder at 12-month follow-up (t2), even after controlling for age, gender and baseline Internet use disorder symptoms. All of the three regression models showed a significant beta coefficient for baseline Internet use disorder. Model 2 and 3 depicted lower age to be also statistically significantly associated with Internet use disorder symptoms 12 months later (see also Table 2). In sensitivity analyses, we also controlled for the treatment conditions to which participants were assigned in the two studies. We found very comparable results for the conditions, indicating that treatment condition did not differentially influence the predictive effects of social anxiety and depression on Internet use symptoms.

**TABLE 2:** Multiple linear regression analyses concerning control variables and predictors of Internet use disorder symptoms.

	Increase in symptoms of Internet use disorder (t2) multiple regression models, standardized β (95% confidence interval; CI)		
Variable	Model 1	Model 2	Model 3
Gender^2^	−0.05 (−2.13; 0.61)	−0.03 (−1.86; 0.85)	−0.05 (−2.16; 0.56)
Age	−0.08 (−0.70; 0.04)	−0.09* (−0.74; 0.00)	−0.09* (−0.74; −0.01)
Internet use disorder symptoms at t1	0.42*** (0.33; 0.52)	0.42*** (0.34; 0.53)	0.40*** (0.32; 0.51)
Depression^1^	0.18*** (1.45; 4.39)	–	0.13** (0.55; 3.71)
Social Anxiety^1^	–	0.19*** (1.61; 4.96)	0.13* (0.56; 4.16)
Adjusted *R*^2^	0.23	0.23	0.24

*^1^Clinically relevant symptoms.*

*^2^Coding: 0 = male, 1 = female.*

*p < 0.05; **p < 0.01; ***p < 0.001.

## Discussion

The aim of this longitudinal study was to investigate, if clinically relevant symptoms of depression and social anxiety predicted future symptoms of Internet use disorders and gain knowledge about possible trajectories that may interconnect both disorders.

This is important because to date, the majority of the few existing longitudinal approaches did not investigate clinically relevant internalizing disorders as predictors of future Internet use disorder symptoms. First studies have identified single items of internalizing symptoms, such as emotional problems, distress or self-esteem problems, to predict future problematic Internet use (e.g., Strittmatter et al., 2016; Wartberg et al., 2021). However, the trajectories of clinically relevant internalizing disorders and behavioral addictions in adolescents have been largely unknown.

In line with our proposed theoretical model of trajectories (see Figure 1), a significant temporal relationship between (a) clinically relevant depression and Internet use disorder symptoms at 12-month follow-up and (b) clinically relevant social anxiety and Internet use disorder symptoms at 12-month follow-up were observed. Moreover, our findings indicate that (c) both trajectories exist independently of each other.

To the best of our knowledge, this is the first study investigating clinically relevant depression and social anxiety in adolescents to predict Internet use disorder symptoms at the 12-month follow-up. There have been previous studies examining associations between internalizing disorders and behavioral addictions in the past. Yet, most of the studies were conducted cross-sectional (e.g., Vadher et al., 2019), and applied screening measures to detect symptoms of depression and social anxiety (Cho et al., 2013; Wartberg et al., 2020). Moreover, most studies were based on adult samples (e.g., Lee and Stapinski, 2012). The study of Ko et al. (2009) was most comparable to our study design as it took both clinically relevant symptoms for social anxiety and depression as predictors of Internet addiction within youths in a longitudinal approach into account. Ko et al. (2009) found – among other psychiatric symptoms - depression and social anxiety to predict the occurrence of Internet addiction in a 2-year follow-up survey among female, but not male adolescents. Thus, this study is partially in line with our results. However, we did not observe such gender effect. Our study supports currently available findings that both genders seem to be at an equal risk of developing Internet use disorders (Wartberg et al., 2021), except for gaming. Furthermore, the study by Ko et al. (2009) was conducted in 2009, before uniform criteria of Internet gaming disorder were published in DSM-5 (American Psychiatric Association, 2013). Another longitudinal approach of Cho et al. (2013) identified symptoms of anxiety and depression in childhood to predict Internet addiction in Korean adolescents, but, the authors used a general screening measurement for psychopathology in childhood and their study population consisted of only male participants, thus limiting possible generalization. In line with our findings, Gámez-Guadix (2014) found that psychological strain predict an increase in components of problematic Internet use. The authors used a similar sample, however, applied a measure for Internet use disorders which was designed in 2010. Even if this scale comes close to the DSM-5 criteria of Internet gaming disorder, it does not obtain full coverage of all nine criteria. In addition, the author did employ a measure which operationalizes psychological strain primarily, containing a subscale which detects depressive symptoms. Our findings go beyond this result and indicate that adolescents with clinically relevant depression show an increased risk to develop future Internet use disorders.

To our knowledge, this is also the first study demonstrating that clinically relevant social anxiety in adolescents increases risk of developing future Internet use disorder symptoms. There are very few studies investigating associations between social anxiety and Internet use disorders independently from depression. All of these studies were conducted as cross-sectional surveys and most of them were based on adult samples (i.e., Lee and Stapinski, 2012; Taranto et al., 2015). As an exception, Vadher et al. (2019) found problematic Internet use to be associated with social anxiety disorder in their cross-sectional study of Indian adolescents. This association has also been found in a German study on adolescent patients (Wartberg and Kammerl, 2020). Our longitudinal findings go beyond these cross-sectional associations and demonstrate a significant relationship between social anxiety and behavioral addictions. As postulated in our theoretical model (see Figure 1), we find indications that social anxiety increases the risk of prioritizing Internet activities and avoiding social activities, which again could reinforce symptoms of Internet use disorders.

The growing relevance of behavioral addictions in childhood and adolescence, most of all, Internet use disorders, is still enhanced since the COVID-19 pandemic changed everyday life profoundly. According to the results of the COPSY-study [German acronym for “*Co*rona und *Psy*che” (Ravens-Sieberer et al., 2021)] the majority of children and adolescents in Germany showed a significant increase in mental health issues during the COVID-19 pandemic, reporting, inter alia, an increase of emotional distress, anxiety and depressive symptoms. In addition, during the COVID-19 pandemic, the amount of time spent online (especially on weekdays) has increased significantly among youths (Dak-Gesundheit (Ed.), 2020; Medienpädagogischer Forschungsverbund Südwest, 2020), increasing the risk of developing a behavioral addiction, such as Internet use disorders (Király et al., 2020; Rumpf et al., 2020). Reasons for this increase may be found in the pandemic related limitation of alternative social and physical activities, which are naturally experienced as rewarding. Based on these aggravations, our findings represent an important insight for researchers, clinicians and any parties involved in the working field of prevention and intervention measures to retain mental health of children and adolescents. The present results stress the importance of identifying adolescents at risk of developing Internet use disorders to guide them to appropriate prevention and intervention measures in timely manner. In the aftermath of the pandemic it appears even more important to sensitize not only professionals working with the concerned risk groups, such as clinical practitioners, psychotherapists, psychologists, and trained specialists for behavioral addictions. It is also crucial to increase awareness about Internet use disorders within families, especially of youths suffering from internalizing disorders so that they may be prevented from going from bad to worse.

### Limitations

There are several limitations of our study that need to be considered. First, we did not confirm clinically relevant depression and social anxiety by clinical interviews to validate diagnoses. Yet, we employed normed and validated questionnaires which are used in the routine diagnostic process within the scope of clinical practice. Furthermore, our results are based on an at-risk sample with a high proportion of participants having clinically relevant symptoms of depression and social anxiety disorder.

Another limitation is that data were aggregated from two prevention and early intervention studies. However, in sensitivity analyses the results were controlled for effects of treatment condition and the findings were very similar for the conditions.

A further limitation includes concerns about the validity of the modified CSAS-J questionnaire. This instrument was constructed and validated for Internet gaming disorder and not validated for our adapted version including both problematic Internet use and Internet gaming disorder. Hence, we could not use standardized CSAS-J values to clinically interpret our outcome. Nonetheless, internal consistency was high in the current study supporting the conclusion that the modified instrument was also a reliable measure of Internet use disorders. In addition, it needs to be mentioned that by adapting the CSAS-J for Internet use in general, we may have captured responses from individuals either referring to both gaming and non-gaming activities combined or referring to each internet activity solely. Yet, this aspect was of minor interest in the present study, as we considered problematic gaming behavior to be part of Internet use disorders. To improve this methodical issue in the future, a different measure of general Internet use could be applied to see if our findings can be replicated. For future research, it may also be interesting to see if internalizing disorders differ in their predictive power regarding non-gaming and gaming internet activities.

Despite the fact that all of our regression models were statistically significant, overall rather low proportions of variance were explained. One reason for this may be drawn from our model of trajectories. We did not examine all potentially relevant mediators such as maladaptive emotional regulation or reward deprivation. Yet we proceeded on the basis of a theory-based model to derive hypotheses for pathomechanisms and trajectories that could interconnect both disorders. Our findings indicate that internalizing disorders increase risk of future behavioral addictions. Based on these results new hypotheses can be generated on the role of common pathomechanisms, such as maladaptive emotional regulation and reward deprivation, which are considered to be relevant in the development and maintenance of behavioral addictions (Lindenberg and Holtmann, in press; Wartberg and Lindenberg, 2020). Future studies should investigate, if these individual factors significantly mediate the relation between internalizing disorders and Internet use disorders and thus contribute to a further explanation of variance. Furthermore, there are several studies that show the importance of externalizing problems for Internet use disorders in addition to internalizing disorders (e.g., Wartberg et al., 2016). A supplementary consideration of externalizing disorders in future studies, could therefore additionally increase the variance explanation in regression models.

Last, interactions would also be conceivable, i.e., that internalizing disorders and Internet use disorders could also influence each other, as indicated by studies investigating problematic gaming (e.g., Wartberg et al., 2018b). We did not investigate such interactions.

## Conclusion

The present study was built on a theory-based approach. We employed clinically relevant measures which identified a high proportion of youth of a large at-risk sample to show clinically relevant symptoms of internalizing disorders (depression and social anxiety). These disorders significantly predicted an increase in symptom severity of Internet use disorders at 12-month follow-up. Based on findings showing that problematic Internet use has increased further during the ongoing COVID-19 pandemic, our findings represent an issue of high relevance for national health care and education policies.

There is international evidence for Internet use disorder to be strongly associated with mental disorders, such as depressive and social anxiety disorders. In the majority of the cases, these disorders will be detected before Internet use disorders in psychological settings (Kewitz et al., 2021). Evidence for this can be drawn from a disparate-picture regarding prevalence estimates of Internet use disorder, suggesting that the majority of patients are being treated for other mental disorders despite suffering from Internet use disorders (Kewitz et al., 2021).

In this current study we identified clinically relevant symptoms of depression and social anxiety to both represent unique risk factors for the development of Internet use disorders in youth. These findings support our theory-based model of trajectories and lead to important implications regarding professionals working with the target group as well as families and individuals concerned. Our results aim to raise awareness for the potential risk individuals suffering from depression and social anxiety are facing. It is to be hoped but remains an open question for future research, if remission of depression and social anxiety can reduce the risk of developing Internet use disorders sustainably when treated in time.

## Data Availability Statement

The raw data supporting the conclusions of this article will be made available by the corresponding author upon request.

## Ethics Statement

The studies involving human participants were reviewed and approved by University of Education Heidelberg. Written informed consent to participate in this study was provided by the participants’ legal guardian/next of kin.

## Author Contributions

KLe: data analysis, literature review, and writing of the first draft and revision of this manuscript. SK and LW: supported data analyses and revised the manuscript. KLi: principle investigator of the PROTECT and PROTECT+ study, funding acquisition, study concept and design, project coordination and supervision, data collection, interpretation of data, literature review, and writing. All authors have read and agreed to the final version of the manuscript.

## Conflict of Interest

The authors declare that the research was conducted in the absence of any commercial or financial relationships that could be construed as a potential conflict of interest.

## Publisher’s Note

All claims expressed in this article are solely those of the authors and do not necessarily represent those of their affiliated organizations, or those of the publisher, the editors and the reviewers. Any product that may be evaluated in this article, or claim that may be made by its manufacturer, is not guaranteed or endorsed by the publisher.
